# Biofiltration of Bioaerosols Emitted from Organic Waste Management Facilities: A Review

**DOI:** 10.3390/microorganisms14050963

**Published:** 2026-04-24

**Authors:** Andrés M. Vélez-Pereira, Pablo Bravo Barra, Yiniva Camargo Caicedo, David J. O’Connor

**Affiliations:** 1 Departamento de Ingeniería Mecánica, Facultad de Ingeniería, Universidad de Tarapacá, Av. 18 de Septiembre 2222, Arica 1000000, Chile; 2 Departamento de Química, Facultad de Ciencias, Universidad de Tarapacá, Arica 1000000, Chile; 3 Programa de Ingeniería Ambiental y Sanitaria, Facultad de Ingeniería, Universidad del Magdalena, Calle 29H3 No. 22-01, Santa Marta 470004, Colombia; 4 Grupo de Investigación en Modelación de Sistemas Ambientales (GIMSA), Facultad de Ingeniería, Universidad del Magdalena, Santa Marta 470004, Colombia; 5 School of Chemical Sciences, Dublin City University, D09 E432 Dublin, Ireland; david.x.oconnor@dcu.ie

**Keywords:** biological aerosol, endotoxins, packing media, emission control, risk mitigation, biofilter design

## Abstract

Bioaerosol emissions from biological treatment processes like composting, livestock operations, and wastewater plants pose notable occupational and environmental health risks. Biofiltration is a common mitigation measure for gaseous pollutants, but its effectiveness in controlling bioaerosols is less studied. This review synthesizes current evidence on biofiltration for the removal of bioaerosols. Findings indicate that biofiltration can significantly reduce emissions from waste-related biological processes, although results vary widely and depend heavily on design and operational factors. In composting, agricultural, and wastewater treatment contexts, fungal bioaerosols are consistently removed with high efficiency, often over 90%. Conversely, bacterial removal shows greater variability, from negligible to above 90%, influenced primarily by airflow rate, bed depth, and media stability. Systems with residence times of tens of seconds and bed depths of at least 1 m tend to reliably reduce bacterial counts, whereas undersized, high-flow systems experience marked efficiency losses. The choice of packing material is also crucial; mature, stable media maintain performance, whereas nutrient-rich or unstable substrates can lead to fungal emissions, turning the biofilter into a secondary source. Data on endotoxin removal are limited and remain insufficient for firm design recommendations. Overall, biofiltration’s effectiveness depends on complex interactions among physical retention, biological stability, and design. These insights emphasize the need for future research to focus on standardized, performance-based design criteria supported by consistent reporting and full-scale validation.

## 1. Introduction

**Figure 1:**
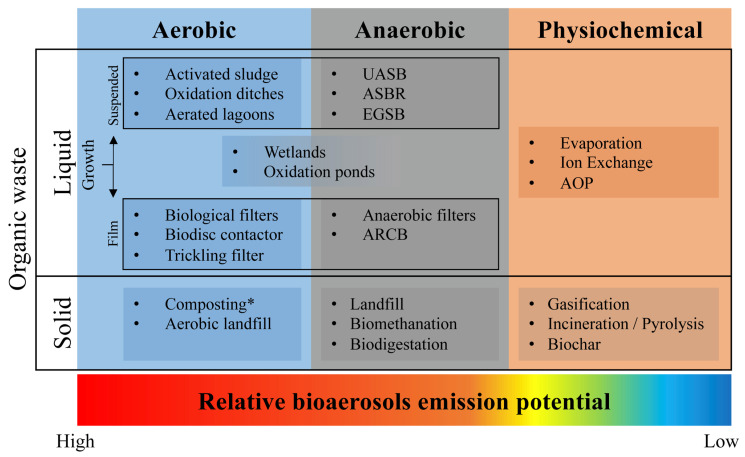
Classification of organic waste treatment systems based on process type (aerobic, anaerobic, and physicochemical) and their relative potential for bioaerosol generation. Liquid and solid waste treatment systems are included. UASB: Upflow Anaerobic Sludge Blanket. ASBR: Anaerobic Sequencing Batch Reactor. EGSB: Expanded Granular Sludge Bed. ARCB: Anaerobic Rotating Biological Contactor. AOP: Advanced Oxidation Processes. * Composting is mainly aerobic, although localized anaerobic conditions may occur within the matrix.

Organic waste management has gained increasing attention due to its significant implications for public health [1]. In this context, biological treatment processes are increasingly recognized as important sources of bioaerosols; therefore, effective practices, including proper handling, inactivation, and disposal of organic waste, are crucial for preventing the spread of disease and reducing environmental pollution [2,3,4]. However, commonly used biological treatment methods can release pathogens and other harmful substances as bioaerosols at various stages of treatment, posing risks to workers and nearby communities [5,6,7,8]. In 2016, global municipal solid waste generation reached 2.01 billion tonnes, with projections indicating an increase to 3.40 billion tonnes by 2050; in low- and middle-income countries, food and green waste constitute over 50% of total waste, whereas high-income countries account for 32% of the total due to greater packaging and other non-organic waste [9]. Meanwhile, worldwide wastewater production is approximately 359.4 billion m^3^ annually, of which 188.1 billion m^3^ are treated [10]. These figures highlight the growing scale of organic waste streams and reinforce the need to understand the generation and control of bioaerosols within these systems. Consequently, the treatment and management of these waste streams are essential from both a public health and environmental standpoint. A variety of technologies are currently used to process both solid and liquid organic waste, each with different implications regarding efficiency and potential health risks. These technologies, including aerobic, anaerobic, and physicochemical processes, are summarized here along with their relative potential for bioaerosol emissions, as shown in Figure 1.

Among the various organic waste management practices, composting and landfilling remain the most widely used systems, despite growing awareness of recycling, which involves recovering materials such as paper, cardboard, glass, metals, textiles, and plastics [11,12]. Aerobic composting primarily focuses on the biological breakdown of organic materials, yielding compost that has numerous beneficial uses in horticulture, agriculture, forestry, soil improvement, and landscape architecture [13]. However, composting and related organic waste management activities are also significant sources of bioaerosols, particularly due to the large amounts of plant residues and animal excreta involved. In composting and organic material recovery, bioaerosol emissions are typically characterized by high fungal and bacterial loads. *Aspergillus* was the most frequently reported, with an average concentration of 267,500 CFU/m^3^. Similarly, bacterial groups, particularly mesophilic bacteria, have been observed at an average concentration of 37,683 CFU/m^3^ [14,15,16]. These observations suggest that bioaerosol emissions and microbial concentrations can vary considerably depending on the characteristics and operational conditions of the treatment process.

In landfills and surrounding areas, fungal genera such as *Cladosporium* have been reported at concentrations up to 19,760 CFU/m^3^, while bacterial genera such as *Bacillus* have been identified at an average concentration of 34,169.61 CFU/m^3^ [17,18,19,20]. In open dumps and municipal landfills, fungal genera such as *Penicillium* have also been detected at high concentrations (4851 CFU/m^3^), whereas bacterial genera such as *Bacillus* continue to be commonly reported, with concentrations around 2851.5 CFU/m^3^ [21,22,23]. These findings indicate that composting and landfill systems are significant sources of airborne microorganisms, particularly fungi and bacteria. However, comparability is limited by variability in sampling protocols, seasonal conditions, and analytical methods across studies, as well as by differences in the type and state of organic waste.

In addition to solid waste systems, wastewater treatment processes are an important source of bioaerosols, particularly from solid-phase byproducts such as sludge. Higher bacterial concentrations are commonly observed in these systems, reflecting the microbial processes that underpin water treatment technologies. One key factor contributing to this is the nature of biological treatment stages, which rely heavily on bacteria specifically adapted to these processes [24,25]. Studies encompassing activated sludge, oxidation ditches, and full-scale treatment systems report that thermophilic fungi were the predominant fungal group, with an average concentration of 872.5 CFU/m^3^, while mesophilic bacteria dominated the bacterial community, reaching up to 17,000 CFU/m^3^ [5,26,27,28,29].

Similarly, in liquid organic waste streams such as domestic or industrial wastewater, elevated bacterial concentrations are frequently observed, often with significant variability. This shift is primarily attributed to the use of microbial consortia tailored to the specific requirements of water treatment technologies [30]. Scientific evidence shows that aerobic wastewater treatment systems emit significantly higher levels of bioaerosols, along with distinct microbial compositions and particle-size distributions, compared with anaerobic systems. This difference is attributed to the presence or absence of oxygen during treatment, although other factors related to the wastewater plant’s design and operating conditions are also involved. Han et al. [5] reported that in an anaerobic–anoxic–oxic treatment system, the highest concentrations of airborne bacteria (1.0 × 10^4^ CFU/m^3^) and fungi (1.44 × 10^4^ CFU/m^3^) occurred in the aerobic tank, while emissions from the anaerobic and anoxic tanks were considerably lower. This disparity arises because aerobic systems incorporate oxygen through submerged aeration, which increases turbulence, promotes bubble formation and bursting, and enhances the air–water interface, all of which facilitate the aerosolization of microorganisms [31,32]. Conversely, anaerobic processes lack aeration and physical agitation, resulting in substantially lower bioaerosol emissions. As an alternative, physicochemical processes are faster, more robust, and efficient, but they involve high operational costs [33]. Despite their potential to emit bioaerosols, biological treatment systems remain favored for their effectiveness in degrading organic pollutants, provided environmental conditions are adequately controlled [34].

Despite the relevance of these emissions, health risk assessments in organic waste decomposition processes have traditionally focused on gases, leachates, and biogas generation rather than on microbial emissions, including bioaerosols [35]. In this context, landfills, the most commonly used method for final disposal of municipal solid waste, have primarily focused on managing biogas and leachate. However, these systems can also create conditions that favor the accumulation and release of high microbiological loads, with bioaerosol emissions reaching up to 10,000 CFU/m^3^. This is largely due to the continuous decomposition of organic matter, the lack of strict controls on bioaerosol emissions, and the greater resistance of fungal spores to deactivation than that of bacteria [36].

**Figure 2:**
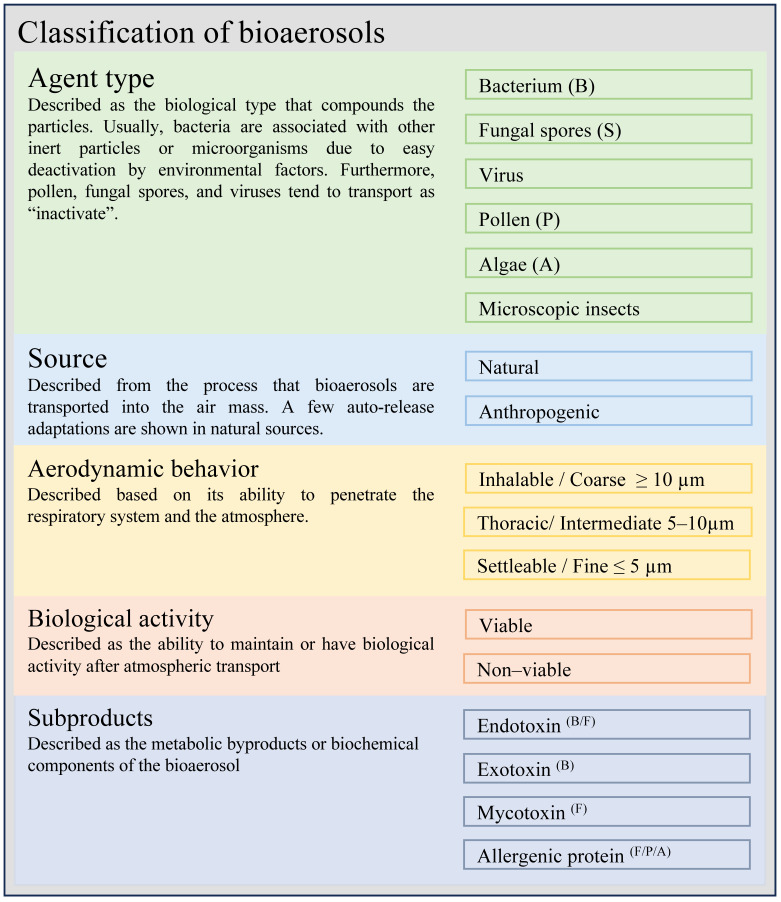
Conceptual classification of bioaerosols based on multiple criteria, including microorganism type, source, aerodynamic behavior, and biological state.

Bioaerosols are airborne particles originating from biological sources, including fungi, bacteria, pollen, viruses, fungal hyphae, and amoebae [37,38,39]. These bioaerosols can have detrimental effects on both human health and the environment [40,41,42]. Their impact depends largely on specific characteristics such as the type of biological agent, viability, metabolic by-products, and emission source, which determine the exposure pathways and the toxicological or immunological responses they may trigger. Bioaerosols can be described using multiple complementary classification criteria rather than a single standardized scheme, as summarized in Figure 2 [43,44]. For instance, fungal spores may be viable or non-viable and can induce respiratory or dermal allergic reactions depending on their biological activity [45], whereas viruses are typically associated with inhalation-related infections [46]. In addition to regulating their potential impact on human health, particle size is closely related to biofiltration processes, as it directly influences deposition mechanisms, retention efficiency, and the overall performance of filtration systems. In this context, smaller particles typically require biofilters with finer pore structures, which are associated with increased operational costs and higher-pressure drops.

**Figure 3:**
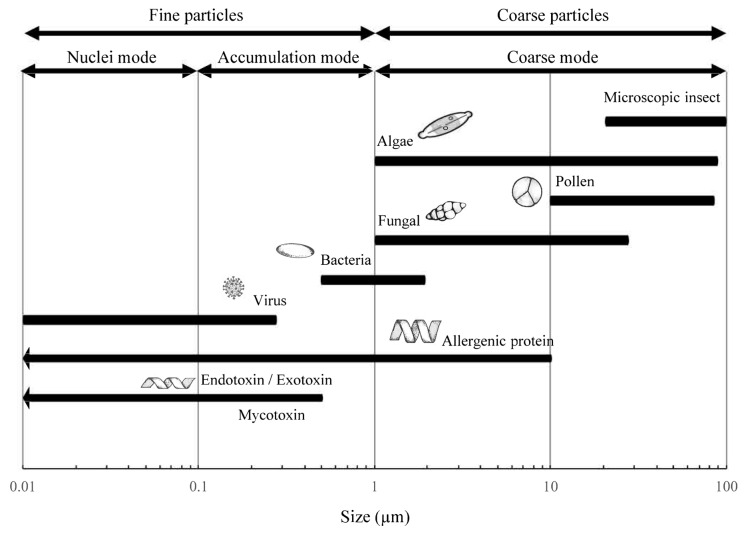
Distribution of aerodynamic diameters according to the type of biological aerosol. Elaboration based on literature [43,44,47,48,49,50,51,52,53,54,55,56,57,58,59,60,61].

Among these characteristics, the aerodynamic diameter is particularly critical (Figure 3), as it determines atmospheric residence time, transport behavior, and the ability of particles to reach the lower respiratory tract, where they can cause more severe health outcomes [44,47,48]. Pollen grains from anemophilous plants represent the largest bioaerosol fraction, with diameters ranging from 17 to 58 μm [49,50]; followed by fungal spores, which range between 1 and 30 μm [51,52,53]; Bacteria typically exhibit smaller diameters, between 0.3 and 3 μm [53,54,55,56]; while viruses are generally smaller than 0.3 μm [57,58]. Within this size spectrum, particles smaller than 5 μm are considered the most critical from a health perspective, as their small size facilitates inhalation and penetration into the pulmonary alveoli, where they may deposit and lead to infections or allergic responses [43,59,60,61].

Among the most commonly reported bioaerosol components, fungal spores and bacteria are of particular relevance in both environmental and occupational exposure scenarios. Fungal genera such as *Cladosporium* and *Aspergillus* are frequently identified and have been associated with health effects, including allergic rhinitis, allergic asthma, and irritation of the skin, eyes, and throat [62,63,64,65,66,67]. Similarly, bacterial genera such as *Bacillus* and *Staphylococcus* have been linked to a range of health outcomes, including skin infections, foodborne illnesses, and severe diseases, such as anthrax caused by *Bacillus anthracis* [68,69,70,71].

Regarding the behavior of bioaerosols in the atmosphere, their resistance to environmental conditions varies among different biological groups, with fungi and viruses generally showing greater resistance to deactivation than bacteria. Across these groups, one of the most critical factors influencing viability is water availability, as it imposes stress on microorganisms, particularly affecting the membrane integrity of bacteria and fungi. At the same time, other agents, such as non-enveloped viruses, may exhibit greater resistance to desiccation [36,72]. Additionally, the ability of bioaerosols to remain suspended in the air, retain viability, and be transported over distances by wind raises significant public health concerns [36]. For this reason, it is essential to assess the factors controlling their emission, dispersion, and survival, particularly those associated with the emission source, as the inherent complexity of these processes and their dependence on the aerobiological approach, especially sampling and quantification techniques, can lead to variable interpretations and complicate the design and implementation of effective control and mitigation strategies.

At present, there are no widely harmonized regulatory frameworks governing routine quantitative bioaerosol monitoring in productive activities, leading to heterogeneous monitoring practices and limited comparability of exposure assessments across studies and regions. However, various control technologies have been developed to reduce airborne contaminants, among which biofiltration has emerged as a relevant approach. Biofiltration systems can serve as effective mitigation strategies, reducing bioaerosols generated during activities that may affect human health and the surrounding environment, while simultaneously contributing to odor control and reducing pollutants such as sulfur oxides, nitrogen oxides, carbon oxides, and volatile organic compounds [73].

Despite these advantages, biofiltration presents several operational limitations, including the need for appropriate selection of filter media according to the target application, periodic replacement of the media, and sensitivity to environmental variables such as temperature and airflow velocity [73]; In parallel, alternative physical and chemical technologies have been explored, including ultraviolet irradiation, thermal inactivation, ionization, and ozonation; however, these methods are often constrained by high operational costs, safety concerns, and variable efficiency [74,75]. Furthermore, certain bioaerosols, particularly bacteria and fungi, may exhibit reduced susceptibility to specific chemical treatments [76,77]; while other approaches require strictly controlled operating conditions; for example, ultraviolet disinfection systems depend on precise exposure geometry and maintenance to ensure effectiveness [78].

In this context, the present review aims to analyze biofiltration processes for bioaerosol emissions from organic waste treatment systems, including both solid and liquid waste streams. Specifically, this article examines the application of biofilters and how their physical and operational characteristics influence performance across different waste and wastewater treatment systems. In addition, the review evaluates reported concentrations of microorganisms, their potential impacts on the health of workers and surrounding communities, and the environmental implications of these emissions. Through this approach, particular attention is given to the underlying mechanisms governing biofiltration performance and the factors that determine its effectiveness under different operational conditions. A detailed characterization of bioaerosol emissions from organic waste treatment facilities is included to provide a necessary framework for contextualizing the design, performance, and limitations of biofiltration systems.

## 2. Biological Aerosols from an Organic Waste Treatment Facility

Bioaerosol emissions from organic waste treatment facilities pose significant environmental and health risks due to their complex composition, high variability, and potential pathogenicity. Understanding their concentration, composition, and dispersion patterns is also essential for evaluating the applicability and performance of biofiltration systems, as these characteristics directly influence removal efficiency and process design. Human activities, particularly in the agricultural sector; composting facilities; sanitary landfills; dumpsites; and wastewater treatment plants have been identified as sources of bioaerosol emissions. In organic waste treatment systems, mechanical handling, aeration, agitation, and material turning are key processes that actively promote the generation and release of bioaerosols. These environments can foster the growth of highly pathogenic and opportunistic microorganisms, which pose health risks to workers [79,80].

Furthermore, there is a risk that these microorganisms could be transported to densely populated areas, such as parks, residential neighborhoods, and commercial districts [30,81,82]. As a result, they may contribute to diseases and environmental issues, including sick building syndrome, viral epidemics, allergy outbreaks during pollination seasons, structural degradation, and contamination of crop plantations [30,79,83,84]. These characteristics highlight the need for effective control and mitigation strategies, among which biofiltration systems have emerged as a promising approach.

The concentrations of viable microorganisms in areas impacted by human activities exhibit significant variability. In the agricultural sector, 682 fungal species have been identified, with *Cladosporium*, *Penicillium*, and *Aspergillus* being the most prevalent [85,86,87,88,89]. Regarding bacterial species, 137 have been reported, with *Staphylococcus* emerging as the most common [86,90]. In urban environments, 34 fungal species were identified, again dominated by *Cladosporium*, *Penicillium*, and *Aspergillus*. Notably, no bacterial species were recorded in this category. At dumpsites, 206 fungal species were identified, with *Cladosporium*, *Penicillium*, and *Aspergillus* once again leading in abundance [20,21,22,38,91,92]. For bacterial species, 69 were noted, with *Bacillus* and *Staphylococcus* the most prevalent [20,38,91,92,93,94].

**Figure 4:**
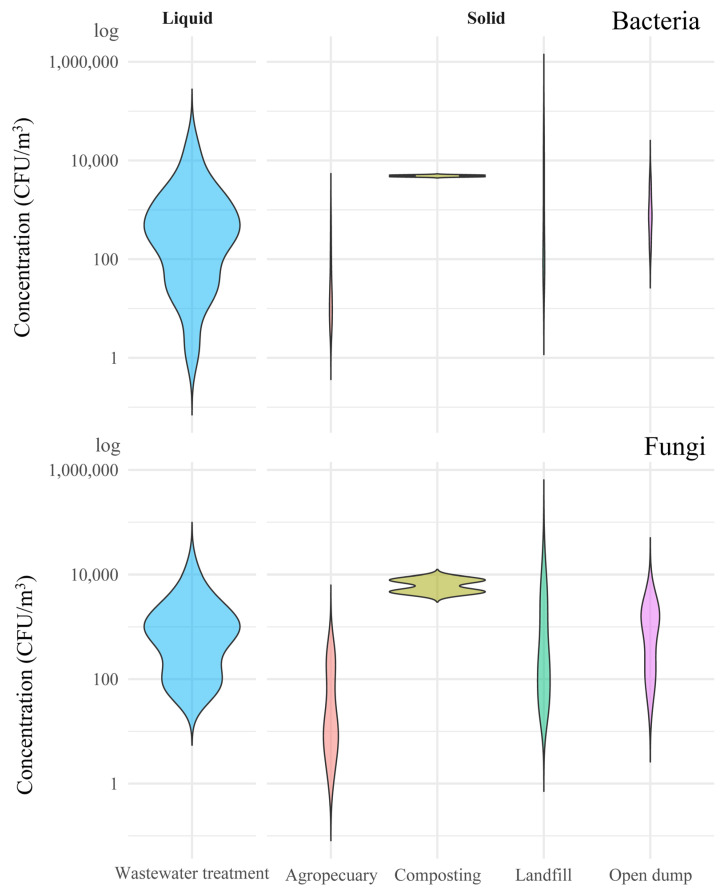
Bioaerosol concentration reported by waste treatment type. (source: [14,17,18,19,20,21,22,23,26,27,28,29,85,86,87,90,98,99,100,101,102,103,104,105,106,107,108,109,110,111]).

In composting facilities, six fungal species have been reported, with *Aspergillus fumigatus* and various yeasts being the most prevalent. Regarding bacterial populations, seven species were identified, predominantly *Staphylococcus* and *Bacillus* [14,15,16,18,95]. In sanitary landfills, greater diversity was observed, with a total of 207 fungal species reported. The highest concentrations were found in *Cladosporium*, *Penicillium*, and *Aspergillus.* For bacteria, 67 species were identified, with *Bacillus* and *Staphylococcus* the most prevalent [17,18,19,20,21,22,23]. In wastewater treatment, several fungal species have been documented, with *Penicillium* emerging as the most dominant; among bacterial species, 19 were reported, with *E. faecalis* being the most common [26,27,28,29]. The concentration of airborne bacteria was consistently higher than that of fungi across all waste facilities (Figure 4). Particularly elevated bacterial loads were observed in organic waste and composting facilities, where values frequently exceeded 10^3^ CFU/m^3^. In contrast, fungal concentrations were comparatively lower and showed greater variability between types, with wastewater treatment areas and landfill facilities exhibiting the highest values. Both bacterial and fungal concentrations varied significantly across treatment locations, suggesting that regional or climatic conditions may modulate bioaerosol emissions [66,96,97].

These factors can modulate the aerobiological spectrum by affecting microbial growth, survival, and sporulation dynamics, as previously demonstrated in fungal communities [112]. In line with earlier observations, facility-specific characteristics such as system design and operation, together with the composition and physical state of the waste, whether liquid or solid, also contribute to shaping the abundance and diversity of airborne microorganisms. It is also important to note that the concentration data presented here are largely derived from culture-based methods expressed in CFU/m^3^; therefore, methodological choices such as sampling device, culture media, and analytical protocols may introduce additional variability beyond that captured in Figure 4. Consequently, part of the observed heterogeneity may reflect differences in measurement approaches rather than true environmental variability, even under comparable treatment systems and operating conditions. In this sense, and as observed in studies of criteria air pollutants, aerobiological research should converge toward standardized methodologies and protocols to ensure comparability across studies.

The direct impact of emissions on nearby communities can occur through two primary pathways. The first is outlined in the study by Sánchez-Monedero et al. [32], which indicates that bioaerosol concentrations did not significantly differ at a reference distance of 200 m, suggesting that this distance is insufficient to reduce microorganism levels to background levels. Even other studies have shown long-range transport from natural fungal spore emissions [97,113,114], which are lower than those from anthropogenic sources such as composting facilities [34,41,94]. In waste-treatment contexts, dispersion is typically dominated by near-field atmospheric dynamics and operational intensity rather than regional-scale transport. The authors also note that their findings align with those reported by the Composting Association in the United Kingdom, which recommends a minimum distance of 250 m. This recommendation is echoed by the Environment Agency, which urges composting facilities to ensure they do not pose adverse health risks to nearby residents, as cited in [95]. However, other studies have recorded air concentrations exceeding background levels at distances of 500 m or more from composting sites [91,115,116,117,118]. Likewise, studies have documented the presence of pathogens emitted from poultry farms at distances greater than 3 km [119,120,121], indicating that the effective radius for the natural decay of bioaerosol concentrations may be significantly greater. The second route of dispersion for biological aerosols generated at waste treatment plants involves vectors, with plant workers being the most significant, followed by nearby communities to a lesser extent [92,94,122,123,124,125,126]. This phenomenon can lead to the emergence of new epidemiological hotspots in areas previously considered to have minimal impact, posing a serious public health concern [94,127,128].

Overall, while the available evidence clearly illustrates the potential for both short- and long-range dispersion of bioaerosols, the definition of a single threshold distance is inherently limited by the biological diversity of emitted particles and their influence on aerodynamic transport behavior. In addition, the detection of bioaerosols at a given distance does not necessarily imply viability, as transport processes may preserve particle presence while reducing their biological activity. Therefore, the assessment of exposure should not rely solely on distance-based criteria but rather on integrated approaches that account for spatiotemporal transport dynamics coupled with biological decay processes, thereby enabling a more accurate representation of viable bioaerosol dispersion under specific environmental and operational conditions [72].

Moreover, Taha et al. [129] point out that open-air compost piles are considered a passive source of aerosol emissions and indicate that agitation or disturbance of the material is not the sole factor responsible for aerosolizing biological materials. They report emission rates for compost piles ranging from 5.9 × 10^6^ to 890 × 10^6^ CFU/s [129,130]. Simultaneously, they describe the decay of concentration as a logarithmic function of distance, reinforcing the conclusions reached by Sánchez-Monedero and Stentiford [95], as well as other researchers [95,117,131,132]. These findings are consistent with Le Goff et al. [133], who reported detectable bioaerosol impact up to 100 m, with concentrations approaching background levels beyond 200 m, depending on emission intensity and wind conditions.

In recent years, many composting processes have shifted to enclosed environments, such as buildings or controlled spaces, when the scale of the managed waste permits [13,134,135]. This approach offers practical advantages in controlling environmental variables, including humidity and temperature, which directly affect the efficiency of the composting process. Additionally, it helps to mitigate, though not eliminate, the problems associated with the emission of unpleasant odors and biological aerosols linked to this activity.

For wastewater treatment plants, recent measurements indicate that culturable bioaerosol concentrations near active treatment units typically range from 10^2^ to 10^4^ CFU/m^3^ for bacteria and fungi [7]. For example, Zhao et al. [136] found that a microporous aeration basin emitted only 20–262 CFU/m^3^ of culturable bacteria (in autumn/winter), roughly an order of magnitude less than mechanical disk aerators. Other studies report peak near-source counts in the low 10^3^ CFU/m^3^ range, e.g., ≈1.5 × 10^3^ CFU/m^3^ in an aerated sludge digester, or median fungal counts ≈6.7 × 10^3^ CFU/m^3^ at some plants [105]. In general, aerated basins and mechanically agitated stages produce the highest emissions, whereas passive processes (trickling filters, clarifiers) yield much lower airborne counts [7,105].

Bioaerosol levels fall off rapidly with distance. Near-field sampling (within ~1–2 m downwind of an aeration tank) can record on the order of 10^4^–10^5^ CFU/m^3^, but at a few meters away, the counts typically drop to 10^1^–10^3^ CFU/m^3^ [123]. For example, one study reported 1.2–8.0 × 10^4^ CFU/m^3^ at ~1–1.5 m from a tank, versus only 2.2 × 10^1^–4.58 × 10^3^ CFU/m^3^ at 2–10 m downwind [123]. Dispersion modeling likewise predicts strong dilution: under typical winds, aerosolized microbes from an aeration basin can be transported over distances of a kilometer or more before falling to near-background levels [137]. Atmospheric factors (wind speed, humidity, turbulence) critically govern this decay. In enclosed or well-ventilated indoor areas (e.g., control rooms, covered tanks), concentrations are generally much lower—often near ambient—levels than on outdoor tank exteriors. Workers in plant buildings are mainly exposed to re-entrained aerosols when ventilation is inadequate, so good filtration and ventilation, along with closed basins, are recommended to minimize indoor levels [123].

No universally prescribed “safe distance” exists for bioaerosols from wastewater treatment plants, but precautionary buffers are often used. For example, the UK Environment Agency’s guidance for waste-processing sites specifies a separation of ~250 m from sensitive receptors (residences, schools), consistent with measurements showing that bioaerosols usually dilute to background levels by a few hundred meters, as cited in [138]. Likewise, planning experts recommend situating major aeration tanks well upwind of populated areas [123,138]. In practice, most field studies find bioaerosol levels near background by a few hundred meters from active aeration basins, implying negligible infection risk beyond that range. In summary, modern data suggest processes in waste facility centers emit on the order of high quantities of bioaerosols [7,35,136], drop by orders of magnitude within tens of meters, and that maintaining buffers on the order of 1 km is considered prudent for nearby populations, as evidenced by campaign sampling and dispersion models of bioaerosols [20,93,123,129]. Finally, modern data indicate that wastewater plant aeration processes release high levels of bioaerosols, which drop by orders of magnitude within tens of meters. Maintaining buffers of about 1000 m is considered prudent for nearby populations [7,123,136,138]. Overall, these findings highlight the high emission intensity and spatial variability of bioaerosols, which must be considered when designing effective control and mitigation strategies.

Monitoring methods for bioaerosols have been comprehensively reviewed elsewhere; thus, only a brief overview is provided here to contextualize recent advances in emission characterization [139,140,141,142]. Conventional approaches, including Andersen cascade impactors and other impaction- and culture-based methods, remain widely used but are limited to discrete sampling intervals and may underestimate short-term variability driven by operational and meteorological factors [142,143]. In contrast, real-time optical instruments based on light-induced fluorescence (LIF) enable continuous detection and classification of airborne biological particles using fluorescence signatures and size information [140]. Systems such as WIBS and its variants (e.g., WIBS-NEO) provide single-particle resolution and have improved the characterization of dynamic emission patterns in both ambient and waste-management environments [142,144,145,146]. These instruments show good agreement with conventional methods while enhancing the observation of short-term dynamics and environmental drivers, although classification uncertainties and fluorescence interference persist under complex field conditions [145,146,147]. Emerging online techniques, including Raman spectroscopy, mass spectrometry, and hybrid optical systems, may further improve analytical specificity [139,140,148]. Overall, these approaches complement conventional methods by improving temporal resolution and supporting a more mechanistic understanding of bioaerosol emissions and their control. Overall, these approaches provide essential tools for characterizing bioaerosol emissions and support the development and optimization of biofiltration systems.

## 3. Impact Associated with Biological Aerosols from the Organic Waste Treatment Facility

Biological aerosol emission, concentration, and accumulation can be positively or negatively influenced by human activities, especially those involving the management of organic materials susceptible to decomposition. These activities can result in the release of highly pathogenic and opportunistic microorganisms into ecosystems and, potentially, into humans. This difficulty arises primarily because the dissemination process usually involves not just a single aerosolized microbial cell, but a consortium of multiple microorganisms contained within a microscopic “mega-particle”, which complicates the identification of opportunistic pathogenic agents and the assessment of associated risks. These health and environmental impacts further underscore the need for effective bioaerosol control strategies, particularly in waste treatment systems.

Additionally, the biological response to a given agent can vary significantly, even at the symptomatic level, within a single species or genus exposed to the pathogen. Furthermore, the existing lack of precise methodologies for accurately determining the viability of microorganisms, from their emission source to potential hosts, poses significant limitations. This shortcoming has led to classifications in the field of aerobiology (viable and non-viable aerosols) that create considerable uncertainty regarding exposure levels and durations to biological aerosols, further complicating the establishment of reliable risk assessments.

There is a well-documented significant association between exposure to elevated levels of bioaerosols and various respiratory pathologies and skin diseases among workers in composting plants [149,150,151]. In some studies, bioaerosol concentrations exceeding 10^4^ CFU/m^3^ have been reported and associated with increased occurrence of respiratory tract irritation [5,152], gastrointestinal issues, and, less frequently, severe respiratory illnesses [34,153,154].

Meteorological conditions undoubtedly play a crucial role in influencing bioaerosol concentrations in the environment [66,136,155,156,157]. So far, ambient air monitoring has been the primary method employed at waste facility centers to evaluate bioaerosol exposures. Recent investigations have been developing a risk assessment [5,136,151,153,158]. These methodologies are based on the EPA chronic daily intake (CDI) and relative risk approaches; however, a more in-depth investigation of the relative risk values is required, as exposure to biological agents is determined not only by concentration levels but also by host-specific and population-level responses. In this context, susceptibility may vary not only between individuals but also across populations due to differences in baseline health status, environmental adaptation, and prior exposure history. Prolonged or repeated exposures may lead to adaptive responses and tolerance in some cases, whereas in others they may increase sensitivity or exacerbate adverse health effects. Moreover, even within the same individual, the response to bioaerosol exposure may vary across the life course, reflecting age-dependent changes in immune function and vulnerability. Furthermore, investigating the impact of process engineering on reducing bioaerosol exposure within facility units is necessary.

In addition to the risk of exposure to biological aerosols, the presence of microbial volatile organic compounds (MVOCs) poses another significant concern [159,160,161,162]. MVOCs are byproducts of the metabolic processes that occur during the decomposition of organic matter by microorganisms. This issue is further complicated by the presence of mycotoxins in fungal aerosols and endo- and exotoxins in bacterial aerosols. There is limited knowledge of the potential adverse health effects associated with occupational exposure, particularly regarding MVOC mixtures found in workplace environments [163]. Only a few studies have reported MVOC concentrations at composting facilities, with levels ranging from 0.01 to 1 mg/m^3^ [157,164,165,166]. However, recent studies show the highest values; for example, Persoons et al. [15] reported values up to 40 mg/m^3^ during the trituration and putrefaction phases. They also report that terpenoids and alcohol are the main volatile compounds, with temperature and turning the compost pile being the main factors affecting MVOCs. In wastewater plants, MOVC emissions range from 0.6 to 134.8 ppm [167] and are produced during digestion and dehydration processes, which emit indoles, mercaptans, and sulfur compounds [168]. These show the necessity of monitoring and protecting workers, especially across waste treatment stages, where temperature, humidity, and agitation affect peak emissions, which occur early and during high microbial activity with poor aeration.

**Figure 5:**
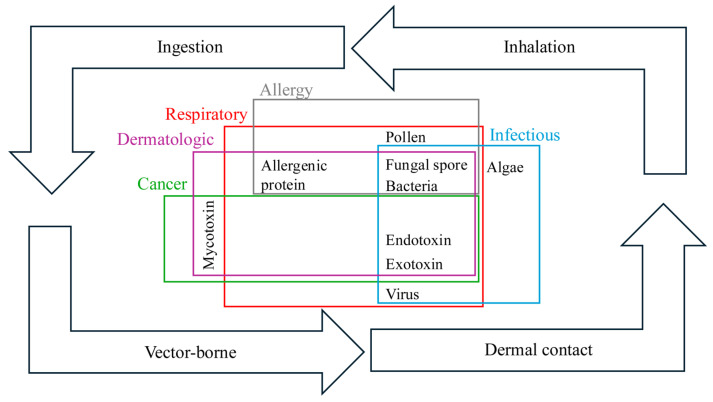
Type of Health Effects by biological aerosols and Routes of Exposure (recreated from Caicedo, 2011).

**Figure 6:**
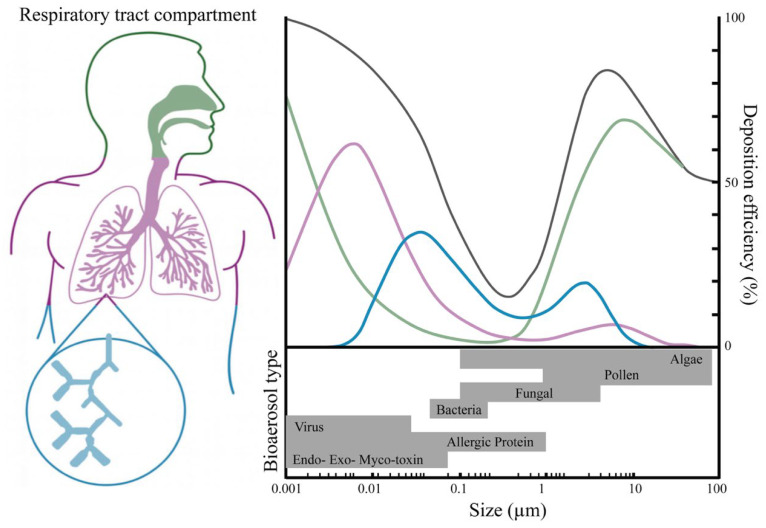
Deposition of Biological Aerosols by Aerodynamic Diameter and Respiratory System Response. Black line: Total deposition curve corrected for inhalability. Green line: Naso-oropharyngeal deposition. Lilac line: Tracheobronchial deposition. Blue line: Pulmonary deposition. (Recreated by the author from Caicedo [176] and Phalen [177]).

Diseases related to exposure to bioaerosols can be divided into five main categories: infectious, respiratory, allergies, dermatologic, and cancer, as shown in Figure 5. However, reliable prevalence data are often lacking in most cases where disease incidence due to biological agents is reported [43]. Human exposure to bioaerosols can occur through inhalation, ingestion, and skin contact, with inhalation being the primary route leading to adverse health effects [60,104,124,169]. Figure 6 shows the system response for elimination based on aerosol deposition and their aerodynamic diameter. This issue is more concerning when examining aerosol concentrations in waste-center facilities, where size-resolved measurements showed the greatest accumulation in the respirable fraction, particularly between 1 and 2.5 µm [16,88,170,171,172,173]. The concern regarding exposure levels is heightened, as particles smaller than 5 µm can penetrate the respiratory system’s defenses and deposit in the pulmonary alveoli, potentially leading to acute respiratory infections. Indeed, several recent studies have documented real-world health impacts of bioaerosol exposure in waste-processing facilities. For example, composting centers and landfills can aerosolize pathogenic fungi and bacteria; prolonged inhalation of Aspergillus fumigatus spores at such sites has led to rare cases of invasive aspergillosis in susceptible individuals [23,34]. Likewise, wastewater treatment plant workers are routinely exposed to airborne opportunistic pathogens (e.g., Legionella, Pseudomonas, Klebsiella, Mycobacterium) that can cause respiratory infections, among other illnesses [79,174]. In addition to these infections, bioaerosols in waste facilities are known to trigger significant allergic and dermatological effects. Occupational exposure to compost dust has been associated with hypersensitivity pneumonitis, chronic bronchitis, asthma, and allergic rhinitis among workers [79]. In these environments, even skin or eye contact with microbial particles or toxins can cause irritation and dermatologic disorders. In fact, studies report higher rates of skin diseases and mucous membrane irritation among compost and waste-handling workers than in unexposed groups [175]. These examples underscore the diverse health risks (from acute infections to allergic reactions and skin inflammation) associated with bioaerosols in waste management settings, highlighting the need for stringent exposure controls and protective measures.

The impacts of bioaerosols on natural environments are varied and largely dependent on their origin, whether from natural phenomena or human activities, as well as the characteristics of the receiving environment. In natural settings such as parks, forests, and grasslands, these impacts can range from changes in microflora, including soil fungi and plant pathogens, to alterations in plant coverage, particularly among species that depend on pollen for reproduction [178]. Moreover, while infrequent, epidemics can occur in populations exposed to specific pathogens. A notable example of this is avian influenza, which is associated with the influenza A virus and its 16 hemagglutinin (H1–H16) and 9 neuraminidase (N1–N9) antigenic variants, collectively constituting the identified species [179].

When examining the situation in urban environments, it is essential to consider the environmental impacts associated with concentration levels and the specific setting, whether indoor or outdoor. In outdoor settings, diseases such as tuberculosis, influenza, and measles can arise from exposure to biological aerosols, including *Aspergillus*, *Cryptococcus*, and *Pneumocystis* species. Furthermore, in natural parks and agricultural areas, these aerosols may adversely affect pollination efficiency [180]. Similar to humans, animals can also suffer the effects of biological aerosols [181,182], potentially developing infectious or allergic diseases, though generally to a lesser extent than in natural environments. Additionally, biological aerosols can cause physical deterioration of infrastructure and monuments through microbial activity [183,184,185,186]. This phenomenon is particularly noted in coastal regions, where marine aerosol-derived microalgae [187,188] coexist with molds such as *Aspergillus* and *Geotrichum* [66,112], originating from natural urban processes. Another physical consequence of biological aerosols is reduced visibility, a phenomenon closely associated with large-scale pollen emissions in temperate regions during spring [189]. In indoor environments, the concentration of bioaerosols is closely linked to health impacts on individuals who reside in or spend significant time in these spaces, such as work or study settings [190]. This phenomenon is often referred to as Sick Building Syndrome, which encompasses a range of illnesses triggered or exacerbated by indoor air pollution [191]. Commonly associated symptoms include allergic reactions, flu-like symptoms, headaches, and irritation. These health issues are frequently accompanied by unpleasant odors closely associated with the specific microorganisms contributing to the syndrome in the building.

While numerous effects of bioaerosols can be observed in various environments, it is essential to recognize that most research has concentrated on outdoor settings, especially those involving agricultural practices, livestock, and animal husbandry, as well as areas affected by organic waste and wastewater treatment processes. Conversely, studies on indoor bioaerosols have primarily focused on healthcare facilities (with a focus on nosocomial infections), public buildings (such as libraries, shopping centers, offices, schools, and health centers), and the food and pharmaceutical industries. Collectively, these findings highlight the importance of implementing efficient mitigation strategies, particularly in environments associated with organic waste management, where biofiltration systems have emerged as a promising approach for reducing bioaerosol exposure.

## 4. Biofiltration of Biological Aerosols

Environmental air pollution management mainly focuses on two strategies: controlling emissions at the source and understanding atmospheric transport to predict pollutant behavior. This approach is because once pollutants are in the atmosphere, dispersion and transformation make control difficult. Research has thus aimed to characterize pollutants, identify sources, quantify emissions, and link them to health and urban impacts. For aerosols, control involves reducing concentration, limiting emission at the source, and curbing dispersion. Significant progress has been made in mechanical particulate removal, with efficient filters, cyclones, and scrubbers now widely used. Specialized filters and adsorbents have also been developed to address challenging emissions, improve fine-particle retention, and reduce airborne contaminants. For aerosols, including marine, biological, inorganic, and organic types, control measures align with the previously mentioned focus, aiming to reduce their concentrations, emissions, and airborne dispersion. Significant advancements have been made, including the design of widely accepted filters and cyclones for sectors that generate particulate matter, as well as the development of specialized filters and adsorbents to improve retention and reduce emissions.

**Figure 7:**
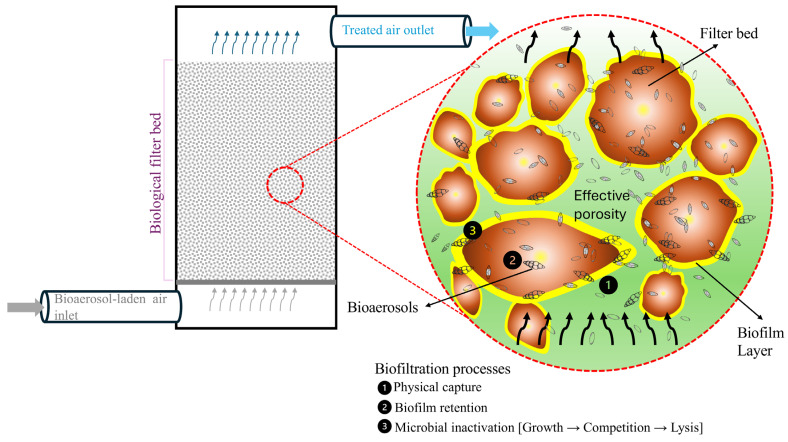
Conceptual representation of bioaerosol removal in a biofiltration system, illustrating airflow through the filter bed and the key mechanisms governing particle retention and transformation within the porous medium.

One control technique of particular interest for bioaerosols is biofiltration, which uses packing materials composed of organic or mixed organic/inorganic substances. In a biofilter, the packing material (often compost, soil, wood chips, peat, or similar substrates) not only physically traps particles but also supports colonies of microorganisms (bacteria and fungi) that actively metabolize or inactivate airborne contaminants (Figure 7). Biofiltration has long been established as an efficient, relatively low-cost, and easy-to-implement technology for controlling atmospheric emissions, especially for malodorous or volatile compounds. It was initially popularized for treating odorous gases from wastewater treatment plants [192,193,194,195,196], sanitary landfills [197,198,199], and agricultural facilities such as animal barns [109,200,201]. Typical biofilters for these gaseous pollutants operate at superficial air velocities of 40–100 m^3^·m^−2^·h^−1^ (with empty-bed residence times of 25–45 s) and can achieve 90–99% removal efficiency for compounds such as hydrogen sulfide, ammonia, and many odorous volatile organic compounds [202,203,204,205]. They typically operate at mesophilic temperatures (around 25–35 °C, optimal for microbial activity) and use packing media such as mature compost, wood chips, bark, or synthetic materials (e.g., polyurethane foam, peat-perlite mixes, or granular cork). These media are selected for their high porosity (60–80%), water-retention capacity, and structural stability, which support a thriving biofilm and efficient gas diffusion [206,207]. Under these conditions, biofilters can sustain contaminant elimination capacities of 60–150 g per m^3^ of filter bed per hour while maintaining >90% removal [204,207,208]. Operational experience shows that these biofilter beds remain effective for roughly 12–24 months (with periodic moisture and pH adjustments) before media replacement or rejuvenation is needed. This robust performance and relatively low maintenance have made biofiltration a widely accepted solution for odor and gas emission control across diverse industries.

Biofiltration is conceptually attractive for bioaerosol emissions because it captures and deactivates airborne microorganisms at the point of emission. The core principles remain similar to those for chemical pollutants, involving air passing through a biologically active porous medium; however, bioaerosol control introduces distinct challenges. The pollutant in this case is living cells or spores and their metabolic byproduct, which can grow or reproduce on the filter medium. Some researchers contend that, due to the presence of these microorganisms, biofilters may not merely act as a control measure for biological aerosol emissions but could also represent a potential source of emissions [39,73,209,210]. In contrast, Tian et al. [211] demonstrate that the relationship between inlet and outlet bioaerosols in full-scale biofilters is not defined by simple microbial transfer but by selective attenuation of health-related risks. Although inlet and outlet bioaerosols share a measurable fraction of microbial taxa, more than 96% of outlet bioaerosols originate from the inlet air rather than from packing materials, indicating that biofilters are not primary sources of emitted microorganisms. Importantly, this taxonomic overlap does not translate into equivalent pathogenic potential. Functional predictions consistently indicate that outlet bioaerosols have significantly lower proportions of pathogenic phenotypes and inferred human pathogens than inlet air, despite only partial community overlap. These results indicate that biofiltration modifies bioaerosols during gas passage, reducing bacterial emissions (5–17%) and disproportionately lowering pathogenic potential relative to total microbial abundance. Thus, Tian et al. [212] provide evidence that full-scale biofilters act as biological risk mitigators rather than amplifiers, even though residual pathogenicity and shifts toward smaller particle sizes highlight the need for complementary upstream control strategies.

Many design and operational considerations overlap with conventional biofiltration. Biofilters used for bioaerosol control generally also require moderate airflow rates (to ensure sufficient contact time), controlled humidity (~40–60% in the bed), and temperatures that support microbial activity without causing desiccation (often maintained in the range of ~15–30 °C, with some studies suggesting the optimal range for bioaerosol filters is 15–22 °C to avoid drying out the bed at higher temperatures; Leson and Winer [213]. In practice, successful bioaerosol biofilters often resemble those used for odor control in construction and materials, with a few modifications to prevent them from becoming new sources of microbes. However, the study proposed shows that the scale-up pathway from the laboratory to full-scale operations underscores why performance is not always transferable. Their results show that laboratory tests indicate that germ removal is strongly influenced by packing type, airflow rate, and moisture content, with coconut fiber showing strong microbial retention under controlled conditions [119]. However, in full-scale open systems, the same sensitivities may be muted, likely because environmental exposure, simplified configurations, and the lack of optimized operational parameters reduce control over key drivers such as humidity distribution and contact time [119]. Even so, fungal reductions above 90% can still be achieved at full scale. In contrast, thermophilic bacteria and actinomycetes may be reduced less effectively, underscoring the need for improvements such as enclosed operation or additional surface layers (e.g., fiber mats) to reproduce laboratory-grade retention under real operating conditions [119].

Research on the biofiltration of bioaerosols is relatively limited compared with the extensive literature on the biofiltration of chemical pollutants. Most published studies on bioaerosol removal via biofilters have been conducted at the laboratory or pilot scale and have predominantly focused on a few key sectors. Early work in this area dates back to the 1990s, exploring biofilters on farm exhaust air [214], and gained greater attention in the 2000s and 2010s as concerns about bioaerosols grew. Notably, Martens et al. [200] conducted one of the first systematic evaluations of different filter media for removing bacteria, fungi, and endotoxins from pig barn exhaust, and Sánchez-Monedero et al. [16] investigated bioaerosol reductions in a composting plant biofilter. Since then, additional studies have examined biofilters in settings such as piggeries and poultry barns [215], composting operations [14,15,79,115,216], waste-handling facilities [217], and wastewater plants [211,216,218,219]. These studies examine a biofilter’s ability to reduce airborne bacteria and fungi, focusing on parameters such as filter bed depth, media type, moisture content, and airflow. Typically, biofilters achieve 70–99% removal of bacteria and spores, but results vary depending on context. Although limited, evidence indicates biofiltration effectively controls bioaerosols in various facilities. Table 1 summarizes biofilter performance by application. The discussion consolidates these findings by source type, then analyzes design factors, filter media effects, endotoxin removal, scale-up challenges, and other considerations.

**Table 1:** Summary of design parameters (volume, bed depth, and packing material), operating conditions (initial bioaerosol load, airflow rate, and temperature), and removal efficiency by agent type for studies applying biofiltration to bioaerosols generated at organic waste management facilities.

	Biofilter Design		Operation Parameter			Bed Composition	Biological Aerosols Removal Efficiency			Reference
	Volume (m^3^)	Depth (m)	Load (CFU/m^3^)	Flow Rate (m^3^/h)	Temperature (°C)		Bacteria	Fungi	Endotoxins	
Agropecuary	1.09	0.5	248,700	0.75	12.6–17.8	Biochips ^1^	69.5 ± 31.6%	69.3 ± 18.6%	96.2 ± 2.26%	[200]
					12.6–17.9	50% Coconut Fiber 50% Peat mixture	8.8 ± 8.2%	74.1 ± 22.7%	98.4 ± 0.9	
					12.6–17.10	50% Chopped bark 50% Fir wood chips	94.9 ± 3.2%	−72.1 ± 210.7%	98.6 ± 1.0%	
					12.6–17.11	33.3% BioContac pellet ^2^ 66.7% Bark	92.4 ± 7%	−408.6 ± 443%	93.1 ± 5.5%	
					12.6–17.12	Crude compost	82.5 ± 22.8%	−99 ± 141.6%	8.7 ± 17%	
	6.48	1.2–1.4	4051.8	1500	N.R	50% Compost 50% Peat	99.6 ± 0.3% *	N.M	11.1 ± 21.2%	[215]
						20% Bentonite 40% Compost 40% Peat	100 ± 0% *	N.M	16.8 ± 32.8%	
						20% Halloysite 40% Compost 40% Peat	100 ± 0% *	N.M	51.5 ± 36.4%	
Composting	1650.0	1.1	110,135	165,000	18–22	Coarse fraction of mature compost Wood chips	89.6% **	99.4% ***	N.M	[16]
	1029.6	1.8	110,135	100,000			39.1% **	97.9% ***	N.M	
	585.0	1.3	110,135	50,000			74.7%**	98.0% ***	N.M	
	132.0	1.2	110,135	16,000			68.1% **	99.3% ***	N.M	
	6.75	1.0	110,135	250			68.1% **	98.7% ***	N.M	
	1680.0	2.4	110,135	70,000		Bark and Pine Roots	88.6% **	90.4% ***	N.M	
	520.0	1.3	110,135	N.R		Peat Pine bark and root	92.2% **	N.R	N.M	
	3.5	1.8	100,000	40.86	N.R	Wood chips	50 ± 85% **	−70 ± 97% ***	N.M	[14]
	110.4	4.6	8145 ^+^	7200	25	40% Compost 50% Return mixture 10% Peanut hull	55.09%	55.59	N.M	[212]
Landfill	1.0	1.2	N.R	40.86	21.2	Polyurethane foam	90%	N.M	N.M	[217]
Wastewater Treatment	0.84	1	3800 ^++^	1.2	21.5	Sawn wood chips	77%	N.M	N.M	[219]
	9		100,000	12,000	60	Air Samples	88%	N.M	N.M	[216]
	52.8	5.6	300 ***	60	N.R	Fiberglass	69.8–98.2%	75.5–99.8%	N.M	[218]
			4765	4320	N.R	Polyurethane Volcanic rock	90.77%	95.86%	N.M	
			4736	5760	N.R		85.73%	91.90%	N.M	
			4585	8640	N.R		79.34%	85.74%	N.M	
	155	N.R	5749	20,000 ^+++^	N.R	Polyurethane Activated sludge	10%	N.M	N.M	[211]
			4635		N.R	Polyurethane Activated sludge Wood chips	17%	N.M	N.M	
	100	N.R	1775	15,000	N.R	Polyurethane Activated sludge Bamboo charcoal	5%	N.M	N.M	
	0.51	2.3	2720–4205	21–81	19–38	Polyurethane Compost (acid pH)	59.24%	N.R.	N.R.	[210]
						Polyurethane Compost (neutral pH)	52.90%	N.R.	N.R.	

* Referred to Gram-negative bacteria. ** Mesophilic bacteria. *** *Aspergillus fumigatus*. ^1^ Reference: Roth GmbH, Oberteuringen, Germany. ^2^ Reference: Krems Chemie AG, Krems/Donau, Austria. + Mean load estimated from the reported range. ++ Load expressed as viable microorganisms per cubic meter. +++ Measured as 16S rRNA gene copies per second, with conversion to cells applied assuming 10% viability/cultivability. Gray cells do not use a biological packing bed; however, they promote biofilm growth that enables biofiltration.

### 4.1. Application of Biofiltration on Bioaeorols Emission of Organic Waste Management Facilities

Biofiltration has been most extensively implemented at composting facilities [14,95,212], where it is primarily used to control odorous emissions and simultaneously reduce the release of biological aerosols generated during organic matter degradation. These systems are typically large, with packing volumes ranging from several hundred to several thousand cubic meters, and they commonly rely on organic media such as mature compost, wood chips, bark, or combinations thereof (Table 1). Across multiple studies, composting biofilters show consistently high removal efficiencies for bioaerosols. Bacterial concentrations are generally reduced by approximately 70–90%, while fungal spore concentrations frequently decrease by 90–99% after treatment. Sánchez-Monedero et al. [16] reported bacterial removal efficiencies of approximately 89–92% and fungal removal efficiencies of 97–99% using a biofilter packed with mature compost mixed with wood chips. Only isolated cases of reduced bacterial performance have been reported, such as a system achieving roughly 39% bacterial removal while maintaining high fungal removal near 98%, which was attributed to specific design or operational limitations. Overall, composting facilities provide strong evidence that biofilters, when designed with sufficient bed depth, adequate moisture control, and stable organic media, can reliably achieve high reductions in bioaerosols, with fungal spores being particularly efficiently captured.

In agricultural and livestock operations, biofilters have been evaluated for treating ventilation air from poultry and swine facilities [200,215]. These systems are generally smaller than those used in composting plants, often consisting of only a few cubic meters of packing material and relatively shallow beds, typically 0.3 to 0.6 m deep, to limit airflow resistance in barn ventilation systems (Table 1). Reported performance in this context is highly variable. Some studies document near-complete bacterial removal, with efficiencies approaching 90–100% [200,215], whereas others report very limited effectiveness, in some cases below 10% [200,211]. Martens et al. [200] demonstrated this variability by testing different packing materials under comparable operating conditions: a spruce bark and wood chip mixture achieved approximately 95% bacterial removal.

In comparison, a coconut fiber-peat mixture achieved only about 9% (Table 1). Fungal aerosol behavior in agricultural biofilters is even more variable. While some systems achieved moderate reductions in fungal levels, others exhibited negative removal efficiencies, indicating net fungal emissions. These negative outcomes were consistently associated with media that supported fungal growth, such as fresh bark or nutrient-rich compost, in which fungal proliferation within the filter led to elevated spore concentrations at the outlet. Martens et al. [200] reported extreme cases in which fungal concentrations increased several-fold, resulting in negative removal efficiencies exceeding 100%. These observations highlight that although biofilters can be effective in agricultural settings, improper media selection or insufficient biological stability can make them secondary sources of bioaerosols. Despite this variability, studies that evaluated endotoxins generally reported high removal efficiencies, often exceeding 90 percent, indicating that biofilters are effective at capturing particulate-associated endotoxins even when fungal control is inconsistent.

Applications at open dumps and landfills are less frequently reported [217], but available evidence suggests promising potential. Li et al. [217] evaluated a compact biofilter with a bed volume of approximately 1 cubic meter and a packing depth of 1.2 m, using polyurethane foam as the filtering medium to treat gas emissions from an open dumpsite (Table 1). Despite its small size, the system achieved approximately 90% bacterial removal. Although fungal concentrations were not reported, this result demonstrates that inert synthetic media can effectively reduce bioaerosol emissions through physical filtration and biofilm formation, even in the absence of nutrient-rich organic substrates. This finding supports the feasibility of using biofilters to improve air quality around landfills, particularly where space constraints limit the use of larger organic beds.

At wastewater treatment plants, biofilters have been implemented to treat air emissions from processes such as aeration tanks and sludge handling, which are known sources of bioaerosols [210,211,216,218,219]. Systems reported in the literature span a wide range of scales, from laboratory units to pilot and full-scale installations treating very large airflows (Table 1). In most cases, well-designed biofilters achieve substantial bioaerosol reduction, with bacterial removal typically ranging from approximately 75–90% and fungal removal often exceeding 85–95%. Liu et al. [218] described a pilot-scale biofilter with a bed volume of 52.8 cubic meters and an unusually deep packing depth of 5.6 m, filled with polyurethane foam and volcanic rock. This system maintained bacterial removal at approximately 79–91% and fungal removal at 86–96% across a range of airflow rates. Similarly, Bélanger Cayouette et al. [216] reported approximately 88% bacterial removal in a full-scale biofilter treating approximately 12,000 cubic meters per hour of air from a wastewater treatment facility. However, not all installations are performed at this level. Tian et al. [211] documented a full-scale system operating at extremely high flow rates, between 15,000 and 20,000 cubic meters per hour, with a relatively limited bed volume. In this case, bacterial removal efficiencies ranged from 5 to 17%, indicating that insufficient residence time and challenges in maintaining effective biofilm activity can severely limit performance. This example underscores the importance of properly scaling the biofilter volume and bed depth to the airflow rate, particularly in wastewater treatment applications where airflow is high. No studies addressing virus removal in biofiltration systems used across different waste management facilities were identified in the literature summarized in Table 1; existing research focuses primarily on bacteria and fungi, with one study on endotoxins.

Taken together, these observations indicate that biofiltration can achieve high removal efficiencies for bacterial and fungal bioaerosols across a range of waste-related activities, but performance is strongly dependent on medium stability, bed depth, and airflow conditions. Compost-based media consistently perform well in composting systems, while agricultural applications require careful control to prevent fungal proliferation. Inert or hybrid media show promise for maintaining stable performance, particularly at landfills and wastewater treatment plants. When adequately designed and operated, biofilters function not only as emission control devices but also as systems that substantially reduce the biological load and associated health risks of air emissions from waste management activities.

### 4.2. Key Design and Operation Factors Affecting the Biofiltration of Bioaerosols from Organic Waste Management Facilities

Across the reviewed studies, biofilter performance for bioaerosol removal shows a clear dependence on physical design parameters and operating conditions, particularly bed depth, airflow rate, and packing material characteristics. The data compiled in Table 1 indicate that bed depths of 1–2 m are most frequently associated with high and stable removal efficiencies, particularly in composting systems. This pattern is well illustrated by Sánchez-Monedero et al. [16], who reported fungal removal efficiencies exceeding 97% and bacterial removal efficiencies between approximately 39% and 90% in full-scale biofilters operating within this depth range. Shallower beds, on the order of 0.5 m, can also perform effectively when airflow is moderate, and the packing medium provides high porosity and biological activity, as demonstrated by Martens et al. [200], who achieved bacterial removal above 90% under these conditions. However, the comparison of studies summarized in Table 1 also indicates that increasing bed depth alone does not guarantee improved performance. Although the deep packed-tower system described by Liu et al. [218] maintained high bacterial and fungal removal at 5.6 m, a compost-based system with a bed depth of approximately 4.6 m reported only moderate removal of both bacteria and fungi [212], indicating that the effect of depth remains conditional on medium stability and overall operational balance.

Airflow rate and the associated empty-bed residence time are equally decisive. The data summarized in Table 1 show a consistent inverse relationship between airflow and bioaerosol removal efficiency across different systems. As airflow increases without a proportional increase in filter volume, removal efficiency declines. This trend is observed across multiple studies compiled in Table 1, and illustrated by Liu et al. [218], where bacterial removal decreased from approximately 91% to 79%, and fungal removal decreased from roughly 96% to 86% as airflow increased and residence time shortened. Similar patterns appear in composting and wastewater treatment applications, where residence times of 30 to 60 s generally correspond to high bioaerosol removal.

In contrast, residence times of only a few seconds result in sharply reduced performance, as documented in the high-flow, full-scale system reported by Tian et al. [211]. Conversely, long residence times achieved through very low airflow can compensate for small reactor volumes, as shown by Soret et al. [219], who obtained substantial bacterial removal in a laboratory-scale biofilter operating at extended contact times. Taken together, these observations indicate that the operational ranges compiled in Table 1 consistently support the need to scale biofilter volume to airflow demand, particularly in full-scale installations handling large exhaust streams.

Operating temperature and moisture conditions further influence removal efficiency by regulating microbial activity and preventing media desiccation. Most systems operate within a mesophilic temperature range, typically 15–35 °C (Table 1), which supports stable biofilm activity while maintaining sufficient moisture in the packing. Across the reviewed systems, stable operation within this temperature range is consistently associated with effective bioaerosol removal, whereas deviations toward drier or hotter conditions tend to reduce performance by limiting microbial activity or promoting media desiccation. Temperatures below this range reduce microbial activity, whereas elevated temperatures can dry the bed and impair performance unless humidification is applied. Consequently, most full-scale biofilters incorporate pre-humidification or water spraying systems to stabilize operating conditions, particularly when treating warm or dry exhaust air. This behavior is consistent with the trends reported by Hu et al. [220], who found that increases in gas temperature and reductions in relative humidity are associated with higher bioaerosol release and reduced mitigation efficiency in gas bioreactors. Consequently, most full-scale biofilters incorporate pre-humidification or water spraying systems to stabilize operating conditions, particularly when treating warm or dry exhaust air.

Among all design variables, the choice of packing material emerges as the most influential factor governing both bacterial and fungal removal. Across the reviewed studies, compost-based media consistently demonstrate the most stable and high bioaerosol removal performance under a wide range of operating conditions. Compost-based media, particularly when mixed with structural amendments such as wood chips, show the most consistent performance across applications (Table 1). These mixtures combine high microbial diversity with adequate porosity and moisture retention, enabling bacterial removal commonly above 80% and fungal removal frequently exceeding 95% [16,216]. In contrast, organic media that lack sufficient structural stability or biological maturity can promote fungal proliferation, resulting in reduced fungal removal efficiency, as observed in several agricultural biofilters reported by Martens et al. [200]. This contrast between stable and unstable organic media highlights a recurrent pattern in the reviewed systems, where nutrient-rich or poorly stabilized materials can shift the filter from a removal system to a secondary bioaerosol source.

Inert and synthetic media, including polyurethane foam and mineral packings, provide an alternative strategy by eliminating intrinsic biological growth while maintaining high surface area and structural stability. Once colonized by biofilm, these media can achieve bacterial and fungal removal comparable to compost-based systems, as shown by Liu et al. [218]. Their lightweight nature allows for deeper reactor designs without compaction, although effective moisture control remains essential. Poor performance observed in certain foam-based systems appears to stem from insufficient residence time or biofilm instability rather than inherent limitations of the medium itself [211].

Hybrid media incorporating mineral additives further illustrate how material selection can enhance performance. This contrast between stable and unstable organic media highlights a recurrent pattern in the reviewed systems, where nutrient-rich or poorly stabilized materials can shift the filter from a removal system to a secondary bioaerosol source. The addition of bentonite or halloysite to compost-peat mixtures substantially increased bacterial removal to near-complete levels and improved endotoxin capture, demonstrating that sorptive capacity and moisture retention can complement biological mechanisms [215]. These results also highlight the importance of material properties in retaining fine particulate fractions, such as endotoxins, which are not always effectively controlled by biological processes alone. Notably, only one of the reviewed studies reported endotoxin removal, underscoring a significant knowledge gap despite the strong particulate-capture performance observed in these systems.

When bacterial and fungal removal are examined separately, bacterial aerosols generally show robust removal across a wide range of media, rarely exhibiting net increases at the outlet. Fungal aerosols, by contrast, are more sensitive to medium composition, with high removal achieved when the packing does not support fungal growth and negative efficiencies occurring when it does. This asymmetry highlights the importance of medium conditioning, biological stability, and competition within the biofilm.

Overall, the collected evidence indicates that high bioaerosol removal efficiency is achieved through an appropriate combination of bed depth, residence time, and carefully selected packing material. Across the reviewed systems, these parameters consistently interact to determine performance, rather than acting independently. Mature compost blended with structural amendments remains the most reliable option, while inert and hybrid media offer promising alternatives when moisture and residence time are adequately controlled. Deviations from these design principles, particularly excessive airflow relative to bed volume or unstable organic media, consistently lead to diminished performance. This synthesis reinforces the view that biofilter efficiency for bioaerosols arises from the balance between physical design and biological stability, as observed across the compiled studies.

### 4.3. Efficiency Performance of the Biofiltration of Bioaerosols from Organic Waste Management Facilities

The differential behavior of bacterial and fungal bioaerosols in biofilters emerges as a consistent pattern across the reviewed studies. Under well-controlled conditions, fungal spores are generally removed with efficiencies comparable to, or higher than, those observed for bacteria. In systems achieving bacterial reductions of approximately 70 to 90%, fungal removal frequently reached values of 95 to 99%. This behavior is consistently observed across different applications and operational conditions, reflecting the larger aerodynamic size of fungal particles (Figure 3), which favors interception and deposition within the filter bed, as well as their limited capacity to proliferate when the packing material is biologically stable. Even in cases where bacterial removal was moderate, fungal control remained high. A representative example is reported by Sánchez-Monedero et al. [16], in which a compost-based biofilter achieved only about 39% bacterial removal while still retaining nearly all fungal spores, indicating that physical filtration alone can be highly effective for larger bioaerosol fractions.

The principal limitation associated with fungal bioaerosol control is not capture efficiency but the risk of net emission when the packing medium promotes fungal growth. Unlike bacteria, which were never observed to increase in concentration across the biofilter in the reviewed studies, fungi showed negative removal efficiencies in several cases. This occurred primarily when fresh or nutrient-rich organic media favored in situ sporulation. Consequently, high fungal removal is conditional on appropriate media selection and management, particularly the use of mature or biologically stabilized materials. When these conditions are met, sustained fungal reductions of 95–99% are commonly reported, which is especially relevant given the allergenic and potentially pathogenic nature of many fungi associated with composting and agricultural environments.

Bacterial removal, while generally high, exhibits greater variability among systems. Reported efficiencies range from low values in under-scaled or poorly conditioned filters to reductions exceeding 90% in optimized configurations. This variability reflects the smaller size of bacterial cells and aggregates, which can penetrate deeper into the filter matrix, as well as their greater resilience within biofilms. Nevertheless, many full-scale biofilters consistently achieve bacterial reductions exceeding 80%, thereby substantially reducing exposure risk. Recent findings further indicate that biofiltration does not simply reduce total bacterial abundance but selectively attenuates health-relevant taxa, suggesting that biofilters function as biological risk modifiers rather than passive sieves.

The removal of endotoxins, although less extensively documented, provides additional insight into the performance of biofilters for fine particulate fractions. Measurements reported by Martens et al. [200] indicate that several organic media achieved endotoxin reductions exceeding 90%, consistent with efficient capture of dust and bacterial debris. In contrast, pure compost beds showed very limited endotoxin removal, suggesting that media structure and particle retention capacity are critical for controlling submicron contaminants. Complementary results from Tymczyna et al. [215] demonstrate that amending compost-based media with mineral clays substantially enhances endotoxin retention, increasing removal from marginal values to approximately half of the inlet concentration. These findings indicate that endotoxin control is strongly medium-dependent and benefits from materials with high surface area and adsorptive properties.

**Figure 8:**
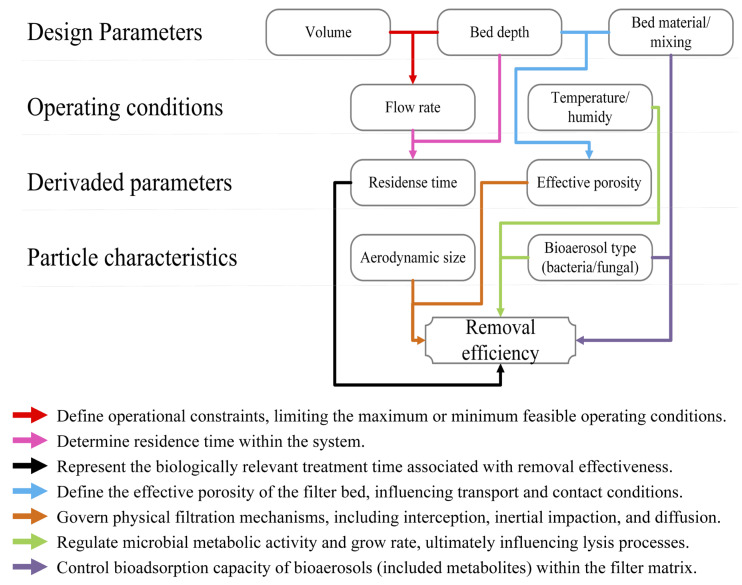
Conceptual framework illustrating the integrated control of bioaerosol removal in biofiltration systems. The diagram highlights how design and operational factors, together with bioaerosol characteristics, influence transport-related conditions and ultimately regulate removal performance through coupled physical and biological processes. Colored arrows represent distinct functional roles of system variables in constraining operating conditions, defining transport dynamics, and modulating physicochemical and biological mechanisms involved in bioaerosol removal.

Taken together, the evidence indicates that biofilters are generally more effective at controlling fungal bioaerosols than bacterial ones, provided that the packing material does not act as a secondary emission source. Bacterial removal is more sensitive to design and operational conditions but remains substantial in well-managed systems. Endotoxin removal, while not universal, appears as an important co-benefit when particulate filtration is effective. Overall, these results reinforce the role of biofiltration as a robust strategy for reducing multiple bioaerosol components simultaneously, with performance governed less by a single mechanism than by the combined effects of media stability, particle size, and filter operation (see Figure 8 for a summary of design–operation interactions governing bioaerosol biofiltration efficiency).

Although biofiltration emerges as a technically robust and economically attractive strategy for bioaerosol control, its current evidence base remains limited compared with the extensive literature on gaseous pollutant removal. This limitation is not only reflected in the relatively small number of full-scale studies addressing bacterial, fungal, and endotoxin removal, but also in the frequent reliance on complementary or alternative technologies to mitigate residual risks. Although viruses are recognized as relevant components of bioaerosols, particularly in wastewater treatment contexts, their removal in biofiltration systems remains poorly understood. The current body of research is largely focused on bacteria and fungi, with limited attention to viral behavior and removal mechanisms. This represents a critical knowledge gap, especially given the potential persistence of non-enveloped viruses under environmental conditions, and highlights the need for targeted studies to evaluate virus fate and removal efficiency in biofiltration systems. As highlighted by Hu et al. [220], physical and chemical disinfection approaches such as ultraviolet irradiation, oxidative agents, photocatalytic systems, plasma processes, microwave treatment, and mechanical filtration have been investigated to address bioaerosol emissions, each introducing specific trade-offs related to energy demand, operational complexity, secondary pollutant formation, or incomplete mitigation of microbial fragments and endotoxins. Importantly, several of these methods effectively inactivate microorganisms without necessarily removing their structural components, thereby maintaining potential inflammatory or toxic effects in the treated air. In this context, biofiltration occupies a distinct position by combining physical retention with biological attenuation, offering the potential to reduce both viable bioaerosols and associated health risks under low-energy conditions. Nevertheless, the limited number of comparative studies, the variability in reported efficiencies, and the frequent need for downstream polishing steps underscore that biofiltration of bioaerosols remains an evolving field. These gaps highlight the need for integrated treatment concepts and for more systematic full-scale evaluations that focus not only on removal efficiency but also on biological stability, pathogenic potential, and long-term operational performance.

### 4.4. Challenges in Performance Interpretation and Design of Bioaerosol Biofilters

The current body of literature on biofiltration of bioaerosols is characterized by high variability in system design, operational conditions, and influent loads, which significantly limits the ability to conduct robust meta-analyses or derive generalized design criteria. This heterogeneity reflects differences in reactor configurations, packing materials, airflow regimes, and waste characteristics, making it difficult to establish consistent performance benchmarks across studies. Moreover, a large proportion of the available evidence is based on laboratory- or pilot-scale systems, which often operate under controlled, simplified conditions. As discussed in previous sections, these systems may not adequately capture the complexity of full-scale operations, where environmental variability, operational fluctuations, and less controlled conditions can lead to markedly different performance outcomes. Consequently, the transferability of results from laboratory and pilot systems to real-scale applications remains limited, highlighting the need for a stronger emphasis on full-scale studies.

In addition, most studies have adopted observational approaches, with limited use of factorial or systematically controlled experimental designs. This restricts the ability to isolate the effects of key operational parameters and to define clear operational boundaries for biofilter performance. As a result, critical factors such as residence time, media characteristics, moisture content, and airflow distribution are often evaluated independently rather than in an integrated manner. Together with the variability in system configurations, this lack of structured experimental design further constrains the development of standardized methodologies and performance criteria.

From a future perspective, recent advances in real-time monitoring techniques offer significant opportunities to overcome some of these limitations. These approaches can improve temporal resolution in bioaerosol measurements and, importantly, enable spatially resolved assessments within full-scale biofilter systems. Such capabilities would support the development of mechanistic and kinetic models describing microbial behavior under dynamic operational conditions, thereby contributing to the identification and optimization of key design parameters. Furthermore, integrating advanced detection techniques may enable the inclusion of additional biological agents, such as viruses and microbial metabolite derivatives, which are currently underrepresented in biofiltration studies. This would not only strengthen the validation of biofiltration as a control strategy but also help delineate its limitations relative to physicochemical technologies, which, despite higher operational costs, may offer advantages in controlling more resistant or non-negotiable biological agents.

## 5. Conclusions

This review demonstrates that biofiltration constitutes a potentially viable strategy for controlling bioaerosol emissions from biological waste treatment processes, particularly when systems are adequately designed and operated. Across composting, agricultural, and wastewater treatment applications, biofilters often achieve high removal of fungal aerosols and variable reductions in airborne bacteria, provided that adequate residence times and biologically stable packing materials are employed. The analysis further highlights that biofilter performance is determined not solely by physical filtration but also by interactions among airflow conditions, microbial dynamics, and medium composition. Importantly, the evidence suggests that biofilters do not inherently act as sources of bioaerosols; rather, undesired emissions may arise from design or operational deficiencies. However, the interpretation of these findings remains constrained by the heterogeneity of experimental conditions and the predominance of laboratory- and pilot-scale studies. Despite the limited number of full-scale studies, the available data provide indicative support, rather than definitive evidence, for the role of biofiltration as a biological risk mitigation technology. Future research should prioritize standardized, health-relevant assessment approaches, including endotoxin- and pathogenicity-related endpoints, and expand full-scale studies to evaluate long-term operational stability and comparative performance under realistic conditions, thereby reducing current uncertainties in performance interpretation and design transferability.

## Data Availability

No new data were created or analyzed in this study. Data sharing does not apply to this article.
